# Cryptogenic hepatitis patients have a higher *Bartonella* sp.-DNA detection in blood and skin samples than patients with non-viral hepatitis of known cause

**DOI:** 10.1371/journal.pntd.0010603

**Published:** 2022-07-18

**Authors:** Marina Rovani Drummond, Luciene Silva dos Santos, Renata Soalheiro Fávaro, Raquel Silveira Bello Stucchi, Ilka de Fátima Santana Ferreira Boin, Paulo Eduardo Neves Ferreira Velho

**Affiliations:** 1 Applied Research in Dermatology and Bartonella Infection Laboratory, University of Campinas, UNICAMP, Campinas, São Paulo, Brazil; 2 Division of Dermatology, Department of Medicine, UNICAMP, Campinas, São Paulo, Brazil; 3 Division of Infectious Diseases, University of Campinas, UNICAMP, Campinas, São Paulo, Brazil; 4 Department of Surgery, University of Campinas, UNICAMP, Campinas, São Paulo, Brazil; University of Utah, UNITED STATES

## Abstract

**Background:**

This study aimed to assess the prevalence of *Bartonella* sp.-DNA detection in blood and skin samples from patients with non-viral end-stage liver disease awaiting liver transplantation.

**Methodology/Principal findings:**

Blood samples and healthy skin fragments from 50 patients were tested using microbiological and molecular methods. Fifteen patients had cryptogenic hepatitis (CH) and 35 had alcoholic, drug-induced or autoimmune liver disease. DNA was extracted from whole blood and liquid culture samples, isolates, and skin fragments. Thirteen of the 50 patients (26%) had *Bartonella henselae* DNA detection in their blood (9/50) and/or skin (5/50) samples. Colonies were isolated in 3/50 (6%) and infection was detected in 7/50 (14%) of the 50 patients. *B*. *henselae-*DNA detection was more prevalent in patients with CH than in other patients (*p* = 0.040). Of 39 patients followed-up for at least two years, a higher mortality rate was observed among patients with CH infected with *B*. *henselae* (*p* = 0.039).

**Conclusions/Significance:**

Further studies assessing the role of *B*. *henselae* infection in the pathogenesis of hepatitis patients must be urgently conducted.

## Author summary

One in four patients with end-stage liver disease awaiting liver transplantation for hepatitis of non-viral origin had documented *B*. *henselae*-DNA detection and cryptogenic hepatitis patients have a higher bacterium molecular detection than patients with non-viral hepatitis of known cause.

## Introduction

In spite of *Bartonella* genus of bacteria being a major cause of emerging and reemerging diseases, these pathogens are still neglected worldwide, even though there has been a recent increase in medically-relevant studies of infections in humans and animals [1]. This genus has a considerable number of species and subspecies, many of which are pathogenic to humans. Infection by *Bartonella* spp. may present different clinical manifestations, potentially fatal, especially in immunodeficient patients who are more susceptible to developing chronic infections [2]. With worldwide distribution and predilection for infecting mammals, many of the *Bartonella* spp. use domestic animals as reservoirs, especially dogs and cats which often become infected by vectors and maintain close contact with pet owners and other humans [3].

A wide spectrum of clinical manifestations has been related to *Bartonella* sp. infection. However, because physicians are not familiar with many of the symptoms, they do not even suspect that these manifestations are caused by these bacteria. Therefore, even if sensitive and specific tests were readily available, they would not be used. Because of this lack of awareness on the part of physicians, patients with diseases previously considered idiopathic or cryptogenic (ie. fever of unknown origin) frequently have the *Bartonella* sp. infection confirmed only after the disease becomes chronic [4,5].

For this reason, the early diagnosis of *Bartonella* sp. infection is extremely important. However, there is no gold standard test to confirm infection by *Bartonella* sp.. Although improvements in diagnosis techniques, such as the use of enrichment media and digital PCR, have increased sensitivity, the possibility of false negative results still exists [6,7]. At least 50 to 100 genome equivalent (GE) may be necessary for molecular detection [8,9] and it is known that bacteremia by Gram-negative bacteria in asymptomatic donors is around 10 GE/mL of blood [10]. Serology also offers limited sensitivity and specificity, depending on technique, while still not ensuring active infection [11]. Bacterial isolation requires special conditions, which are not available in routine hospital laboratories, and it can take up to six weeks for these microorganisms to grow on a solid culture medium [4]. Histopathological analyses are useful with tissue other than blood have injuries, but this methodology has insufficient sensitivity to detect the bacteria, even when silver staining and immunohistochemistry techniques are used. On the other hand, molecular tests may be more sensitive in these tissues even when there are no lesions [12].

The conditions that have been associated with infection by *Bartonella* sp. includes fevers of unknown origins, recurrent or severe anemia, hepatitis, serositis, chronic lymphadenopathy, chronic fatigue, uveitis, retinitis, neuritis, febrile maculopapular exanthem, purpura, urticaria, erythema nodosum, erythema multiforme, erythema marginatum, granuloma annulare, and leukocytoclastic vasculitis [2,5]. However, many individuals infected with these bacteria are asymptomatic [13]. A study identified six out of 500 blood donors (1.2%) from University of Campinas (UNICAMP) Blood Center with documented *Bartonella henselae* bacteremia [14].

*Bartonella* sp. can cause angioproliferative lesions, resulting from the angiogenic response determined by bacteria, such as those observed in human skin in patients with bacillary angiomatosis or Carrion’s disease. They can also cause granulomatous processes due to the pattern of chronic inflammation, often seen in lymph nodes and occasionally in the skin of patients with cat-scratch fever (CSD) [15]. In addition to the skin and lymph nodes, several other organs can be affected, such as liver, spleen, bone marrow and the central nervous system. However, infection caused by these bacteria may not promote injury in the mentioned organs, as seen in Oroya fever or trench fever [15].

Hepatic involvement in the infection caused by *Bartonella* spp. is more common in immunodeficient patients, but it can compromise immunocompetent patients, as well [16–18]. Studies have demonstrated that *B*. *henselae* can cause non-specific liver inflammation in both adults and children; granulomatous hepatitis with or without necrosis; hepatic bacillary angiomatosis and peliosis [18–21]. Hepatic involvement is reported in 1 to 2% of cases of CSD, being the third most common clinical manifestation, after fever and lymphadenopathy [22].

*Bartonella* sp. infection has been associated with patients of alcoholic liver disease [23] and liver transplanted patients (LTP) [24]. LTP may have been infected before the transplant or through donated liver or blood transfusion [17,25]. In the United States, solid organ transplant recipients are not encouraged to have direct contact with pets [26]. *Bartonella* spp. can co-infect patients with viral hepatitis B or C and, eventually, may be related to hepatitis recurrence after liver transplantation (LT) or *de novo* hepatitis in these patients [18,27].

Cryptogenic hepatitis (CH), or hepatitis of unknown causes, is defined as a disease that has unexplained by conventional clinical, laboratory and histological findings [28]. It is not an independent disease entity and may have an infectious origin, among other causes. The inability to detect some agents may make it difficult to define CH etiology. This hepatitis may or may not progress to cryptogenic cirrhosis. Cirrhosis is caused by liver inflammation and between 5 to 30% of patients with this diagnosis have a CH. Three to 14% of adults and 22% of children waiting for LT are diagnosed with CH [28]. Of all children with CH, 86% require LT [27,29]. A small proportion of patients awaiting LT have CH at the UNICAMP Clinic Hospital [30].

According to Czaja’s review, recurrence of the hepatitis occurred in 23–54% of LTP diagnosed with CH. This author showed these individuals present low survival rates (one to five years) and, even in transplants due to chronic hepatitis C, alcoholic liver disease and drug-induced fulminant hepatic failure, CH was observed in the post-transplant period [28].

A significant number of patients are currently being listed for LT with a diagnosis of cryptogenic cirrhosis. A study, using a large data set from United Network for Organ Sharing (UNOS), showed that patients with cryptogenic cirrhosis are clinically distinct from nonalcoholic steatohepatitis, alcoholic cirrhosis, or autoimmune hepatitis, but they have similar short and long-term LT survival [31].

Primary LT is a complex and expensive procedure [32]. *Bartonella* sp. infection, when undiagnosed, can potentially influence the survival of the transplant recipient population, especially in those with CH [33]. The histological characteristics of CH usually indicate mild inflammatory activity with or without cirrhosis, some of which are found in liver diseases caused by *B*. *henselae* [34].

No study has systematically investigated the possibility of *Bartonella* sp. as one of the causes of CH. Therefore, this study aimed to compare the prevalence of *Bartonella* sp. in patients with CH and non-viral hepatitis of known cause and to describe retrospectively patients’ clinical courses with a minimum follow-up of two years.

## Methods

### Ethics statement

The project was approved by the University of Campinas Institutional Research Board (CAAE: 10731712.6.0000.5404) and all participants signed an informed consent form.

From May 2016 to March 2017, fifty patients with non-viral hepatitis and end-stage disease who were awaiting liver transplantation, accompanied by the UNICAMP Clinic Hospital, were invited to participate in a program to investigate *Bartonella* sp. infection from their blood and skin samples. They were grouped into patients with CH and patients with non-viral hepatitis of known causes.

Each patient answered a questionnaire to collect personal and clinical data and received information about risk exposure for infection by these bacteria. From each patient, a 10 mL blood sample was collected by venipuncture in tubes containing EDTA. A 0.4 cm diameter fragment of healthy skin was also collected from the internal face of the forearm of each patient, preferably from the left forearm. Skin samples were used since some studies have shown a better sensitivity in diagnosis by PCR using DNA extracted from skin fragment than from blood sample [35–37].

Blood and skin samples were stored at -20°C until the moment of analysis. For *Bartonella* sp. growth in liquid culture, a specific liquid medium was used [38]. After freezing for at least 24 hours, 1 mL of whole blood was introduced into 4 mL of liquid medium. The cultures remained in a constant agitation of 35°C with 5% CO_2_ for 14 days. After this period, 1 mL of the liquid culture suspension was sown in solid medium, prepared according to the previous description [9].

The solid culture was then incubated at 35°C with 5% CO_2_ in an atmosphere saturated with water, for up to 42 days. Growth was assessed on a weekly basis. When observing characteristic growth of the studied bacteria, colonies were collected and stained with Gram stain. If the morphology was suggestive of the genus (small and delicate Gram-negative bacteria), samples of the isolates were collected and frozen for subsequent molecular analysis.

Negative controls were added to ensure the sterility of the culture media and the environments to which the cultures were submitted. These controls were maintained during the entire process (incubation, extraction, and molecular analysis) along with the other samples and consisted of culture media without inoculum.

DNA was extracted from blood samples, liquid blood cultures, solid culture *Bartonella* sp. suggestive colonies, and skin fragments using QI*Aamp* DNA Mini Kit (Qiagen). All extracted samples except for those from bacterial colonies were tested through PCR to amplify a constitutive gene. The gene region selected for the test was a fragment of *GAPDH* (glyceraldehyde-3-phosphate dehydrogenase), an enzyme related to glycolysis and expressed by all mammalian cells [39]. This reaction assesses the quality of extracted DNA and certifies the absence of amplification inhibitors.

All samples were tested by PCR for the presence of *Bartonella* sp. DNA. Three types of PCR were used: conventional PCR, nested PCR and qualitative real-time PCR.

Negative controls (reagents and water, used instead of DNA) were added to all reactions. In addition to these controls, a known concentration of *B*. *henselae* DNA was serially diluted from 100 to 1 genome equivalent (GE) per microliter to determine the sensitivity of the PCR assay. The control DNA was extracted from colonies isolated from cat blood and deposited at the Adolfo Lutz Culture Collection in Brazil under accession number IAL 3715 [9].

In conventional PCR, primers for the genus *Bartonella* were used, targeting the intergenic spacer region (*ITS*) 16S-23S of RNAr [40]. The detection limit for this reaction was 50 GE of *B*. *henselae* per reaction tube, and the expected amplicon length was 604 base pairs (bp). In nested PCR, primers were used, targeting the region that encodes the *FtsZ* protein involved in bacterial cell division. These primers are species-specific for *B*. *henselae* [41]. The detection limit for this reaction was 10 GE of *B*. *henselae* per reaction tube, and the expected amplicon length was 218 bp. For real-time PCR, primers were used to amplify the citrate synthase (*gltA*) region in the SYBR Green system specific to *B*. *henselae*. In this study, real-time PCR results were used as qualitative PCR, considering results as positive or negative. This strategy has already been used by Allizond *et al*. [42]. Amplification was confirmed by electrophoresis; the detection limit for this reaction was 10 GE of *B*. *henselae* per reaction tube, and the expected amplicon length was 191 bp [43].

All PCR products (including real-time PCR) were analyzed by electrophoresis on 2% agarose gel stained with GelRed and visualized under ultraviolet light. The amplicon samples with sufficient quantity and quality were sent for Sanger sequencing.

After collecting blood and skin samples from the 50th patient, a follow-up of the patients was performed for 24 months. Once the presence of *Bartonella* sp. DNA was demonstrated, all live patients with this condition were summoned. They were evaluated and a doctor explained that *B*. *henselae* infection had already been detected not only in patients with liver disease but also in asymptomatic subjects as blood donors. Oral treatment with 300 mg/day of rifampicin and 500 mg of clarithromycin every twelve hours for six weeks was offered to all infected patients.

A statistical analysis was performed to compare and analyze the detections of *Bartonella* sp. DNA in the group of CH patients with the group of non-viral hepatitis of known cause patients. The exploratory analysis used summary measures (mean, standard deviation, minimum, median, maximum, frequency, and percentage). The groups were compared using the Mann-Whitney test, Chi-square test or Fisher’s exact test. The level of significance was 5%.

## Results

Of all 50 patients, 15 had CH and 35 had hepatitis of a known cause (31 with alcoholic hepatitis, two with drug-induced hepatitis, and two with autoimmune hepatitis). The two groups were similar in terms of sex, age, rural or urban area, animal contact (especially cats and dogs), history of animal scratch/bite, insect bite, blood transfusion, and Model for End-Stage Liver Disease (MELD) score.

The results obtained from blood and skin samples showed *B*. *henselae*-DNA in 26% (13/50) of the patients on the liver transplant waiting list, 10% (5/50) of healthy skin fragments and 18% (9/50) of blood samples (two from whole blood, four from liquid culture and three from solid cultures). One patient had *Bartonella* sp.-DNA detection in both blood and skin samples. *B*. *henselae* infection could be documented in seven patients, either by isolation (11, 16, and 20) or by detection of DNA in liquid culture in patients in whom DNA had not been detected in whole blood (15, 25, 34 and 35). All 13 patients with *Bartonella* sp.-DNA detection were identified in *B*. *henselae* species-specific reactions. Of these, seven were patients with CH and six with alcoholic hepatitis. Amplicons from seven patients could be sequenced, confirming *B*. *henselae* detection (Bartonella henselae strain Houston-I chromosome, complete genome. GenBank accession number: CP020742.1).

Table 1 shows the descriptive analysis and comparisons of clinical data in relation to the hepatitis etiology and *Bartonella* sp.-DNA detection. There was a difference only in the comparison of *Bartonella* sp. detection: patients with CH (7/15) had a higher occurrence than the group of those with known etiology (6/35), *p* = 0.040.

**Table 1:** Descriptive analysis and comparisons of personal and clinical data in relation to hepatitis etiology and *Bartonella* sp.-DNA detection.

Patients analyzed	Etiology		*p* -value
	Cryptogenic hepatitis	Other	
(N = 50)	(N = 15)	(N = 35)	
Age (mean ± SD (N))	60.3 ± 7.9 (15)	55.7 ± 8.1 (35)	0.0550^1^
Age (median (min-max))	62.0 (46.0–71.0)	56.0 (36.0–73.0)	
**Sex**			
Female	4 (26.7%)	5 (14.3%)	0.4234^3^
Male	11 (73.3%)	30 (85.7%)	
**Residential area**			
Rural	1 (6.7%)	2 (5.7%)	1.0000^3^
Urban	14 (93.3%)	33 (94.3%)	
**Contact with animals**			
No	1 (6.7%)	4 (11.4%)	1.0000^3^
Yes	14 (93.3%)	31 (88.6%)	
**Dog**			
No	1 (7.7%)	3 (10.0%)	1.0000^3^
Yes	12 (92.3%)	27 (90.0%)	
**Cat**			
No	6 (66.7%)	9 (50.0%)	0.6828^3^
Yes	3 (33.3%)	9 (50.0%)	
**Animal bite/scratch**			
No	13 (92.9%)	28 (84.8%)	0.6532^3^
Yes	1 (7.1%)	5 (15.2%)	
**Insect bite**			
No	2 (13.3%)	6 (17.1%)	1.0000^3^
Yes	13 (86.7%)	29 (82.9%)	
**Blood transfusion**			
No	7 (46.7%)	11 (32.4%)	0.3381^2^
Yes	8 (53.3%)	23 (67.6%)	
**Blood donation**			
No	10 (66.7%)	17 (50.0%)	0.2797^2^
Yes	5 (33.3%)	17 (50.0%)	
**MELD score**			
MELD score (mean ± SD (N))	15.8 ± 3.9 (14)	15.7 ± 3.0 (35)	0.9378^1^
MELD score (median (min-max))	16.0 (8.0–24.0)	16.0 (10.0–23.0)	
***Bartonella* sp.-DNA detection**			
No	8 (53.3%)	29 (82.9%)	0.0403^3^
Yes	7 (46.7%)	6 (17.1%)	

Legend:

MELD: Model for End-Stage Liver Disease

^1^ based on Mann-Whitney test

^2^ based on Chi-square test

^3^ based on Fisher’s exact test

Six of the 50 patients were receiving antibiotics at the time of sample collection. Three had CH (one was receiving ciprofloxacin and metronidazole and two were receiving norfloxacin) and showed *B*. *henselae*-DNA, one of them in both blood and skin samples. Of the patients with non-cryptogenic hepatitis, three were receiving norfloxacin and *B*. *henselae*-DNA was not detected. Table 2 shows a summary of patient data and *B*. *henselae* detection results.

**Table 2:** Data from patients with non-viral hepatitis and detectable *Bartonella henselae*-DNA by PCR.

Patient number	Hepatitis etiology	*B*. *henselae* DNA detection by PCR		Antibiotics use at sample collection	Liver transplant	Treatment after *Bartonella* sp. detection	Death
		Sample	PCR (region)				
10	Alcoholic	Whole blood	Real-time (*gltA*)	No	No	No	Yes
11	Alcoholic	Solid culture	Nested (*ftsZ*)*	No	No	Yes	No
15	Cryptogenic	Liquid Culture	Nested (*ftsZ*)*	Yes	No	No	Yes
16	Alcoholic	Solid culture	Nested (*ftsZ*)*	No	No	No	Yes
20	Cryptogenic	Solid culture	Real-time (*gltA*)	Yes	No	No	Yes
21	Cryptogenic	Skin	Nested (*ftsZ*)	No	No	No	Yes
25	Cryptogenic	Liquid Culture	Real-time (*gltA*)	No	No	Yes	Yes
34	Alcoholic	Liquid Culture	Real-time (*gltA*)*	No	No	No	No
35	Cryptogenic	Liquid Culture	Real-time (*gltA*) and Nested (*ftsZ*)	No	No	No	Yes
39	Cryptogenic	Whole blood Skin	Nested (*ftsZ*)* Nested (*ftsZ*)	Yes	No	No	Yes
45	Alcoholic	Skin	Real-time (*gltA*)	No	No	Yes	No
46	Alcoholic	Skin	Nested (*ftsZ*)*	No	No	No	No
47	Cryptogenic	Skin	Real-time (*gltA*)*	No	Yes	Yes	No

* Confirmed by sequencing (Bartonella henselae strain Houston-I chromosome, complete genome.

GenBank accession number: CP020742.1)

Table 3 shows etiology and evolution after a minimum follow-up of 24 months for patients with non-viral hepatitis with and without detectable *B*. *henselae*-DNA.

**Table 3:** Etiology and evolution after at least two years of follow-up of patients with non-viral hepatitis with and without detectable *Bartonella henselae*-DNA.

Etiology		Total number of patients	*B*. *henselae*-DNA detection by PCR	Evolution		
				Alive	Dead	Unknown life situation
Cryptogenic		15	7	5	10	0
Non-cryptogenic	Alcoholic	31	6	11	12	8
	Drug-induced	2	0	0	0	2
	Autoimmune	2	0	0	1	1

**Fig 1:**
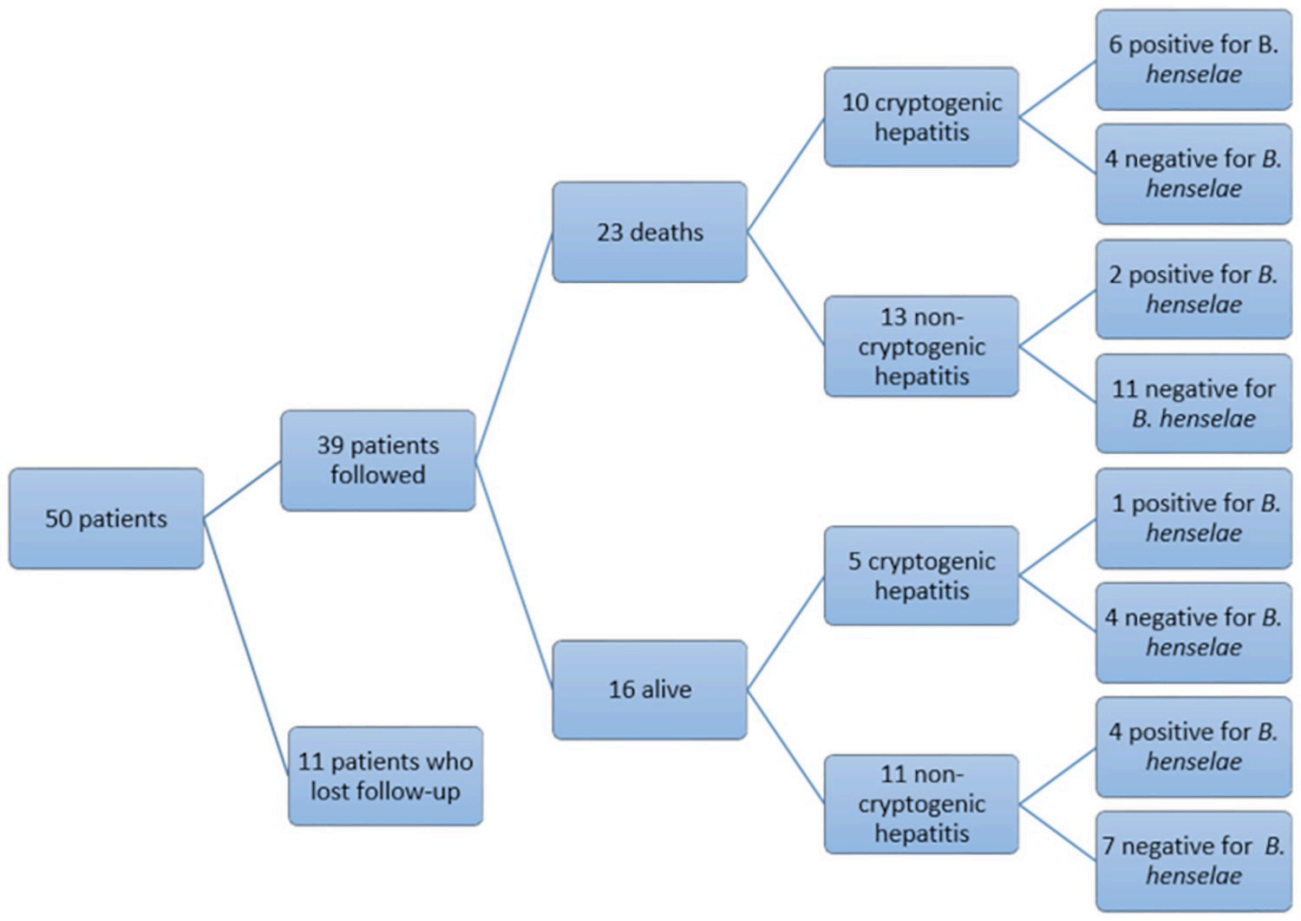
Evolution of the study of patients two years after the inclusion of the 50^th^ patient (non-cryptogenic: Alcoholic, drug-induced, and autoimmune).

Fig 1 shows the evolution of patients after at least two years. Of all 39 patients with known evolution, no statistical difference was observed when comparing patients with CH versus patients with another etiology (*p* = 0.157).

Of these 39 patients, 23 died during a follow-up of at least two years. Among these, ten had CH and 13 hepatitis of known etiology. Eight of the 23 (34.78%) deceased patients demonstrated *B*. *henselae* DNA (six of the eight patients had CH and two had alcoholic hepatitis). Of the other 15 deceased patients, four had CH and 11 had hepatitis of known etiology. A higher mortality rate was observed among patients with CH infected with *B*. *henselae* (*p* = 0.039).

Of all 16 patients who were known to be alive two years after sample collection from the 50^th^ patient included in the study, five demonstrated *B*. *henselae*-DNA (four had alcoholic hepatitis and one had CH and had been transplanted). In the remaining 11 patients, *B*. *henselae* infection was not documented and, of these, four had CH and seven had hepatitis of known cause.

Contact was initiated with the 13 patients with detectable *B*. *henselae*-DNA: five were deceased, two could not be contacted and six were alive and responded. These six patients received information about their infection and about the occurrence of *Bartonella* sp. DNA detection in patients with hepatitis and in asymptomatic blood donors. The oral treatment with rifampicin 300 mg/d and clarithromycin 500 mg every 12 hours for six weeks were offered. Two patients with CH were treated (one was transplanted, asymptomatic and used the antibiotics, and the other presented temporary clinical improvement of cryptogenic peritonitis but died of sepsis ten months later). Four patients with alcoholic hepatitis received the prescription; two were treated with the described antibiotics and were asymptomatic after a minimum follow-up of 24 months. The other two chose to not treat and were also asymptomatic after the same follow-up.

## Discussion

The UNICAMP Clinic Hospital, where the study was conducted, is one of just nine health institutions in Brazil qualified to perform highly complex liver transplants [44]. According to the Brazilian Association of Organ Transplants (*Associação Brasileira de Transplantes de Órgãos—ABTO*), the rate of LT grew by 15.4% between 2012 and 2018 and remains the second most common solid organ transplant in the country. Brazil is the second country in the world in absolute number of liver transplants, behind only the United States, but it ranks 21^st^ in number per million of the population. With 5,192 Brazilian patients with end-stage liver disease awaiting transplantation in 2018, 2,182 LT were performed in the country. The annual cost of this transplant was estimated to be twice the cost of kidney transplantation, the most transplanted solid organ in the Brazil [45].

*B*. *henselae* has been linked with granulomatous liver manifestations, as observed in atypical cases of CSD, including transplanted patients [19,20,22,46], and angioproliferative reactions, seen in angiomatosis and bacillary peliosis [47]. This species has also been described as causing nonspecific hepatitis [19–21], like those observed in many patients analyzed in this study. Acute/subacute liver involvement has been widely described in pediatric and adult, immunocompetent and immunodeficient *Bartonella* sp. infected patients [48]. The authors are not aware of any cross-sectional study analyzing *Bartonella* sp. infection in patients with chronic liver diseases.

This study found a high occurrence of *B*. *henselae*-DNA detection in patients with liver disease of non-viral origin who were in the liver transplant waiting list (26%). The occurrence of the DNA detection and mortality of infected patients were significantly higher in patients with CH. These data reinforce that the detection of *Bartonella* sp.-DNA may influence the survival of patients with CH [33]. Even without statistical difference, the group of patients with CH had a higher median age, which may be related to the higher mortality observed among patients in this group. Although *B*. *henselae* co-infection and the hepatitis B and C viruses have already been reported [27,29], it is assumed that the difference in the bacterium detection would be even greater among CH patients if patients with viral hepatitis were included in the study.

*B*. *henselae* infection could be documented in 54% (7/13) of patients with detectable *B*. *henselae*-DNA. It continues to be necessary to assess the relevance of *Bartonella* sp.-DNA detection in the pathogenesis of CH. Once etiopathogenic involvement is proven, infection treatment for patients with liver disease may prevent progression to cirrhosis or recurrent infection of transplanted patients. Scolfaro *et al*. have already considered donor-recipient bartonellosis transmission in a solid organ transplant [17], but liver donors should also be tested for *Bartonella* sp. infection. Although no standard protocols currently exist, at least PCR tests from blood and liver could be performed. Prophylactic treatment with antibiotics for organ recipients from donors infected by the bacteria may also decrease the occurrence of post-transplant CH in these individuals.

The number of positive results from skin samples (5/13) confirm that this tissue can be used more frequently in *Bartonella* sp. diagnosis, as suggested by other studies [35,37]. *Bartonella* sp. is intraendothelial pathogen and its bacteremia is usually cyclic [14]. There is no single test with enough sensitivity to minimize false-negative results and a test platform is needed for accurate test results.

Bacteremia was confirmed by agent isolation in 3/50 patients (6%). *B*. *henselae* is a fastidious bacterium that does not grow in routine diagnostic culture media. Its primary isolation is difficult, even in proper conditions [3,49]. The detection of patient isolates was compared with that of 500 blood donors in a study conducted at the Blood Center at the same university. In the study with blood donors, enrichment culture was also used in liquid medium before sowing in solid medium. Six isolates (1.2%) were obtained from these asymptomatic individuals [14]. The difference between patients with liver disease and blood donors suggests a higher level of bacteremia in the blood of the patients.

The DNA of *B*. *henselae* could be detected in seven of the 15 patients with CH. One of these seven patients was transplanted a few days after blood and skin samples were collected for *Bartonella* sp. investigation. The explanted liver unfortunately was not evaluated for *Bartonella* sp. infection because there were no results about *B*. *henselae* DNA detection at that time.

Of the 50 patients in the study, six were taking antibiotics at the time of sample collection. The administration of ciprofloxacin or norfloxacin did not prevent the detection of *B*. *henselae* DNA in the molecular tests performed on three patients with CH, one of them with an isolate in solid culture and who had *B*. *henselae* DNA detection in blood and skin samples. *Bartonella* sp. isolation was already obtained in patients under antibiotics [50,51]. Three other patients who were taking norfloxacin and who had liver disease of known etiology tested negative for the infection.

No therapy regimen has been defined in prospective studies for the treatment of patients with hepatitis and *Bartonella* sp.-DNA detection. Spach and Kaplan, 2019, suggest an association of azithromycin and rifampicin for the treatment of an atypical hepatosplenic form of CSD [52]. The same authors present clarithromycin as an alternative to azithromycin. A study analyzing *in vitro* susceptibility of 31 strains of *Bartonella* spp., including 21 strains of *B*. *henselae* and other species, showed that these bacteria were highly susceptible to rifampicin, doxycycline and macrolides, particularly clarithromycin [53]. An association of clarithromycin and rifampicin was already described in a child with hepatic involvement, with echocardiographic improvement of the lesions after two months [54]. Although the treatment time for typical CSD is shorter [55], potentially more serious diseases must be treated for at least six weeks, as indicated for endocarditis caused by *Bartonella* spp. [56]. Oral antibiotic treatment with clarithromycin and rifampicin for six weeks were offered to the patients in this study. The purpose of this study was not to evaluate the effectiveness of the treatment result; however, of four treated patients, three with alcoholic liver disease were alive while one patient with treated CH died after a minimum follow-up of 24 months.

This study was carried out within some limitations: patient follow-up was not an objective of the initial project. Therefore, it was performed retrospectively, after obtaining the results. Patients with *Bartonella* sp.-DNA detection could not be admitted for intravenous treatment with gentamicin. In addition, there is no gold standard diagnostic method with high sensitivity for *Bartonella* spp., which can lead to false-negative result.

One in four patients with end-stage liver disease awaiting transplantation for hepatitis of non-viral origin had documented *B*. *henselae*-DNA detection. The detection was more prevalent in patients with CH. A higher mortality rate was observed among patients with CH with bacterium DNA detection than among the patients with hepatitis of a known cause and the same *B*. *henselae*-DNA detection. The bacterium infection was documented in 14% of patients and *B*. *henselae*-bacteremia in 6%. Further studies assessing the role of *B*. *henselae* infection in the pathogenesis or progress of hepatitis patients must be urgently conducted.
